# Effects of Musical Tempo on Musicians’ and Non-musicians’ Emotional Experience When Listening to Music

**DOI:** 10.3389/fpsyg.2018.02118

**Published:** 2018-11-13

**Authors:** Ying Liu, Guangyuan Liu, Dongtao Wei, Qiang Li, Guangjie Yuan, Shifu Wu, Gaoyuan Wang, Xingcong Zhao

**Affiliations:** ^1^Faculty of Psychology, Southwest University, Chongqing, China; ^2^Institute of Affective Computing and Information Processing, Southwest University, Chongqing, China; ^3^Chongqing Brain Science Collaborative Innovation Center, Southwest University, Chongqing, China; ^4^School of Electronic and Information Engineering of Southwest University, Chongqing, China; ^5^Chongqing Key Laboratory of Non-linear Circuit and Intelligent Information Processing, Southwest University, Chongqing, China; ^6^School of Music, Southwest University, Chongqing, China

**Keywords:** music tempo, musician, non-musician, emotion, fMRI

## Abstract

Tempo is an important musical element that affects human’s emotional processes when listening to music. However, it remains unclear how tempo and training affect individuals’ emotional experience of music. To explore the neural underpinnings of the effects of tempo on music-evoked emotion, music with fast, medium, and slow tempi were collected to compare differences in emotional responses using functional magnetic resonance imaging (fMRI) of neural activity between musicians and non-musicians. Behaviorally, musicians perceived higher valence in fast music than did non-musicians. The main effects of musicians and non-musicians and tempo were significant, and a near significant interaction between group and tempo was found. In the arousal dimension, the mean score of medium-tempo music was the highest among the three kinds; in the valence dimension, the mean scores decreased in order from fast music, medium music, to slow music. Functional analyses revealed that the neural activation of musicians was stronger than those of non-musicians in the left inferior parietal lobe (IPL). A comparison of tempi showed a stronger activation from fast music than slow music in the bilateral superior temporal gyrus (STG), which provided corresponding neural evidence for the highest valence reported by participants for fast music. Medium music showed stronger activation than slow music in the right Heschl’s gyrus (HG), right middle temporal gyrus (MTG), right posterior cingulate cortex (PCC), right precuneus, right IPL, and left STG. Importantly, this study confirmed and explained the connection between music tempo and emotional experiences, and their interaction with individuals’ musical training.

## Highlights

- Fast music evoked positive emotional valence with activation in the bilateral STG.
- Medium music evoked the strongest emotional arousal and lowest emotional valence.
- Medium music activated right HG, MTG, cingulate gyrus, precuneus, IPL, and left STG.
- Musical training led to differentiated neural activation in the left IPL.

## Introduction

As a basic temporal concept in beat perception, tempo is primary importance in determining listeners’ music-evoked emotion (Hevner, 1937; Melvin, 1964; Gabrielsson and Juslin, 2003; Gagnon and Peretz, 2003) and is also an important feature for recognizing different emotional states based on the long-term modulation spectrum analysis (Shi et al., 2006).

Music with different tempi can evoke different emotions. Music with a fast tempo has been found to evoke positive emotions, such as happiness, excitement, delight, and liveliness, while music with a slow tempo evokes negative emotions, such as sadness, depression, and gravity (Peretz et al., 1998; Balkwill and Thompson, 1999; Juslin and Sloboda, 2001). In comparison, speech, as another type of acoustic cue, produces almost the opposite emotional effect. Fast speech has been judged as being less pleasant than slow speech (Ilie and Thompson, 2006). It is thus worth investigating what the neural connections are between musical tempi and their emotional function in listening to music, which can provide a valuable description of music-evoked emotion and promote the application of tempo in regulating music-evoked emotion.

By detecting individuals’ electroencephalogram frequencies that correspond to the tempo of music, researchers have found that musical stimuli with different tempi entrained neural changes in the motor and auditory cortices, which was most prominent in the alpha (8–12 Hz) and beta (12.5–18 Hz) ranges (Yuan et al., 2009; Nicolaou et al., 2017). When listening to highly arousing, usually fast music, alpha activities have been found to decrease in the frontal and temporal areas (Basar et al., 1999; Ting et al., 2007), and beta waves have been detected to increase in the left temporal lobe and motor area (Höller et al., 2012; Gentry et al., 2013). In a functional magnetic resonance imaging (fMRI) study, music with positive emotions was associated with large activation in the auditory cortices, motor area, and limbic systems (Brattico et al., 2011; Koelsch et al., 2013; Park et al., 2014; Bogert et al., 2016). However, no studies have directly compared the differential neural activities of music-evoked emotion aroused by different music tempi, which is a basic and important acoustic feature in music listening. It can be hypothesized that a positive emotional experience when listening to fast music would arouse strong neural activities in the auditory and motor cortices.

Music with a slow tempo often evokes negative emotions (Balkwill and Thompson, 1999; Cai and Pan, 2007; Hunter et al., 2010); however, the neural mechanism behind this is still unclear. Humans do not always prefer slow to fast tempo, while nonhuman primates have been found to prefer music with a slow tempo that was similar to their alarm calls of short broadband bursts repeated at very high rates (McDermott and Hauser, 2007), which implies that humans’ emotional experience of music may be the result of a matched-degree between the music tempo and physiological rhythmic features. During sports training, slow music was found to decrease players’ feelings of revitalization and positive engagement (Szabo and Hoban, 2004). Emotional and psychophysiological research had proved that a decrease in tempo led to a decrease in reported arousal and tension and a decrease in heart rate variability (Van der Zwaag et al., 2011; Karageorghis and Jones, 2014). Therefore, the mechanism of slow music’s emotional effect may be the result of its slowed acoustical arousal compared with humans’ physiological rhythms, such as heart rate or walking pace tempo, which may produce negative emotional effects with weak activation in the auditory cortex.

Few studies have looked at medium-tempo music. Medium music reduced the rating of perceived exertion (Silva et al., 2016) and was preferred at low and moderate intensities of exercise (Karageorghis et al., 2006), which reflected its advantage in optimizing listeners’ physiological load and may be connected with autonomic response. Due to the similarity between medium tempo and humans’ physiological rhythms (∼75 bpm), it could be assumed that it may be easier to process activities in the autonomic emotional network with medium-tempo music than with music of other tempi.

Additionally, tempo has been found to be connected to individuals’ musical training through enhanced activation in the dorsal premotor cortex, prefrontal cortex (Kim et al., 2004; Chen et al., 2008), and subcortical systems (Strait et al., 2009). Compared with non-musicians, musicians were more sophisticated in their recognition of music emotion with stronger activation of the frontal theta and alpha (Nolden et al., 2017), as well as stronger activation in the auditory system through a complex network that covered the cortical and sub-cortical areas (Peretz and Zatorre, 2005; Levitin and Tirovolas, 2009; Levitin, 2012; Zatorre, 2015). Conversely, by examining musicians and non-musicians’ responses to phase and tempo perturbations, researchers found that the ability of individuals to perceive the beat and rhythm of a musical piece was independent of prior musical training (Large et al., 2002; Geiser et al., 2009). Whether the way in which tempo interacts with individuals’ emotional values is dependent upon their musical training has been a controversial question and is worth studying.

In order to investigate the effects of tempo on how humans’ emotional experiences interact with musical training, this study attempted to explore the differences in the behavioral and neural emotional activity induced by music of different tempi between musicians and non-musicians. Typically, tempo is measured according to beats per minute (bpm) and is divided into prestissimo (>200 bpm), presto (168–200 bpm), allegro (120–168 bpm), moderato (108–120 bpm), andante (76–108 bpm), adagio (66–76 bpm), larghetto (60–66 bpm), and largo (40–60 bpm) (Fernández-Sotos et al., 2016). Valence and arousal were chosen as the basic dimensions for detecting listeners’ emotional experience; these were the most popular features in presenting humans’ emotional experiences while listening to music (Bradley and Lang, 1994). First, in this experiment, music with a fast tempo (>120 bpm, presto and allegro), medium tempo (76–120 bpm, moderato and andante), and slow tempo (60–76 bpm, adagio and larghetto) were chosen to stimulate music-evoked emotion. Second, to ensure a neural activation closest to the natural experience of music listening and to avoid the neural activity of a motoric task, we collected the brain activation during a passive music listening activity without any other body movement or cognitive process. Third, to collect individuals’ evaluations of music-evoked emotion, we arranged the subjective emotion assessment of the valence and arousal dimensions soon after the fMRI scanning.

We hypothesized that musicians would show stronger emotional experiences and neural activities than non-musicians. Music with a fast tempo would arouse the most positive emotional reactions with activation in the temporal and motor processing areas. Music with a medium tempo, which is close to humans’ physiological rhythms, would arouse a strong emotional response by entraining the autonomic neural activation of emotion processes. Music with a slow tempo would receive the lowest emotional valence and the weakest emotional arousal by the participants.

## Materials and Methods

### Participants

Forty-eight healthy adult volunteers (mean age 20.77 ± 1.87 years, 25 women, all right-handed) took part in the study after providing written informed consent. All volunteers were free of contraindications for MRI scanning. Twenty-one were musicians, who had at least 7 years of musical experience, either in vocal or instrumental music. Twenty-seven participants were non-musicians, who had no more than 3 years of musical training, other than general education classes before high school. None of the participants in either group had prior experience with a task similar to that used in the present study. They had no history of hearing loss, neurological or psychiatric disorders, and were not taking any prescription drugs or alcohol at the time of the experiment. They all received 50 RMB after the experiment. The study was approved by the Ethics Committee of Southwest University in accordance with the Code of Ethics of the World Medical Association (Declaration of Helsinki). The privacy rights of all participants were observed and protected.

### Stimuli

Instrumental music with three kinds of tempo (fast tempo: >120 bpm, presto and allegro; medium tempo: 76–120 bpm, moderato and andante; and slow tempo: 60–76 bpm, adagio and larghetto) was selected by three music professors. Each tempo group contained 10 songs. These musical excerpts were all serious non-vocal music, chosen to illustrate significant emotions and be representative of the most important instrumental groups (e.g., solo, chamber, and orchestra music). One month before the experimental fMRI scanning, the 30 songs were rated according to valence, arousal, and familiarity by another 15 musicians and 20 non-musicians. Individuals were categorized as musicians if they had at least 7 years of musical experience, either in vocal or instrumental music, and individuals were non-musicians if they had no more than 3 years of musical training, other than general education classes before high school. During the assessment, each rater was sitting in a separate quiet room with a Dell computer, playing music through a set of headphones. After listening to a whole series of music at about 60 dB, raters were required to score the valence, arousal, and familiarity separately on three 7-point scales (1 = very negative, low arousal, unfamiliar and 7 = very positive, high arousal, familiar). Then, twelve music of equal familiarity, with higher arousal in same tempo or with more positive/negative valence for each tempo group, were selected for the formal experiment (Supplementary Table S1). Each group contained four Chinese and non-Chinese non-vocal musical compositions. The first 60 to 90 s of the selected 12 musical pieces were adapted to fit the system requirements of E-prime 2.0 and given 1.5 s fade-in and fade-out ramps.

### Experiment

Before the fMRI experiment, participants were asked to remain calm and complete a practice activity outside the scanner room, which lasted about 1 min. In the practice activity, participants were required to listen to three music clips of 20 s and detect a specific *click-click* sound that lasted 2 s with 0.25 s fade-in and fade-out ramps among them, without missing any. In the formal experiment, this was designed to prevent participants from being inattentive or falling asleep in the scanner.

**FIGURE 1:**
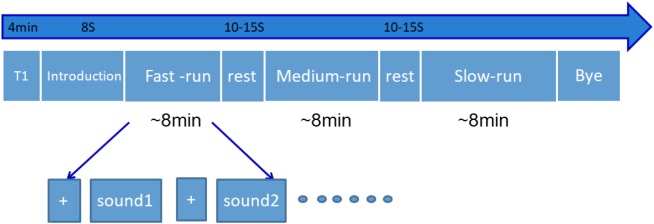
The scanning procedure of the fMRI experiment.

All the participants received a fMRI scan in this experiment. The design for the procedure is displayed in Figure 1. Three runs of fast-, medium-, and slow-tempo music were pseudo-randomly presented among different participants. Each run contained four musical excerpts of the same tempo classification and two detection *click-click* sounds, which were played randomly at five 4-s intervals. The detection sound would be presented at the beginning of each run. Between the runs, participants could rest for 10–15 s. During the scanning, all participants were asked to relax, not to move during the scanning process, and to listen to the music attentively with their eyes closed. Once the detection sound appeared, participants needed to respond by pressing a box button. Meanwhile, their response was recorded by the experimenter outside the scanning room. If the participant missed one detection sound during a run, they would be reminded to concentrate during the rest period. After the scan, the participants were interviewed and all participants except two reported the experiment as being relaxing and stress-free. The scanning lasted approximately 28 min.

The post-ratings of valence and arousal for the same 12 music pieces were performed soon after the scanning. Only after the participant understood the meaning of the two dimensions (valence, the amount of pleasure experienced, which fluctuates from negative to positive, and arousal, the autonomic reaction associated with an experience, which fluctuates from weak to strong) (Liu et al., 2016), could the participant begin the post-rating experiment. The two participants who reported experiencing stress after the fMRI scan finished their behavioral ratings on the day after their scans. During the post-rating, after one musical piece was played, participants were required to rate the music pieces within 4 s for both the arousal and valence dimensions, using two 7-point scales (0 = very negative or low arousal and 7 = very positive or high arousal) based on their subjective music-evoked emotion. The behavioral ratings were completed in a specially organized room, where each participant was placed in front of a computer. Each participant was asked to judge each music piece, which was played on the computer at a volume of 60 decibels (dB). Two intervals of 20–30 s each occurred pseudo-randomly for participants to relax. The post-rating lasted about 30 min. The statistical analysis was conducted using SPSS 20.0. Before and after the neural and behavioral experiments, the State-Trait Anxiety Inventory was used to evaluate individuals’ state anxiety (Spielberger et al., 1970).

### fMRI Acquisition

Images were acquired with a Siemens 3T scanner (Siemens Magnetom Trio TIM, Erlangen, Germany). An echo-planar imaging sequence was used for image collection, and T2-weighted images were recorded per run [TE = 30 ms; TR = 2000 ms; flip angle = 90°; field of view (FOV) = 220 mm × 220 mm; matrix size = 64 × 64; 32 interleaved 3 mm-thick slices; in-plane resolution = 3.4 mm × 3.4 mm; interslice skip = 0.99 mm]. T1-weighted images were collected with a total of 176 slices at a thickness of 1 mm and in-plane resolution of 0.98 mm × 0.98 mm (TR = 1900 ms; TE = 2.52 ms; flip angle = 90°; FOV = 250 mm × 250 mm). We used SPM8 (Welcome Department of Cognitive Neurology, London, United Kingdom)^1^ to preprocess the functional images. Slice order was corrected by slice timing correction, and the data were realigned to estimate and modify the six parameters of head movement. In order to collect magnet-steady images, the first 10 images were deleted. The images were normalized to Montreal Neurological Institute space in 3 mm × 3 mm × 3 mm voxel sizes then spatially smoothed with a Gaussian kernel. The full width at half maximum was specified as 6 mm × 6 mm × 6 mm.

Then we obtained six direction parameters of head movement. We deleted data of participants whose head-movement parameters were more than 2.5 mm. After the preprocessing, data for 7 participants were deleted and data for 41 participants were retained; that is, the final sample consisted of 16 musicians and 25 non-musicians. Two levels of data analysis procedures were used to analyze fMRI data. At the first (subject) level, four event types were defined, which consisted of fast-, medium-, and slow-tempo trials, and detection response. The onset time was chosen when the target sounds were presented. At the second (group) level, full factorial analysis of variance was chosen for a comparison of the three sound conditions between the two groups. To determine whether there was significant activation corresponding to each contrast in tempo, a false discovery rate (FDR) corrected *p* = 0.05 and an extent threshold of cluster size = 20 voxels for the height (intensity) were used as the threshold.

### Stimuli Presentation During the Scanning

The task was programmed using E-prime 2.0 on a Dell computer. Binaural auditory stimuli were presented using a custom-built magnet-compatible system that attenuated around 28 dB. During the scanning, the loudness levels at the head of the participant were approximately 98 dB. After the attenuation of the listening device, the auditory stimuli were approximately 70 dB at the participant’s ears. During a pretest scan, the optimal listening level for each participant was guaranteed by being determined individually.

## Results

### Behavioral Analysis

On the State-Trait Anxiety Scale, the mean trait anxiety score was 52.15 ± 3.08 (range 45–60), and the scores demonstrated a normal distribution. The mean pre-test score was 47.74 ± 6.94, and the mean post-test score was 49.37 ± 6.36, which was consistent with normed values for Chinese college students (45.31 ± 11.99) and confirmed that all participants were in an emotionally stable state (Li and Qian, 1995). The difference between pre- and post-scores was not significant (*p* = 0.65). Internal consistency coefficient (alpha) was 0.93.

**FIGURE 2:**
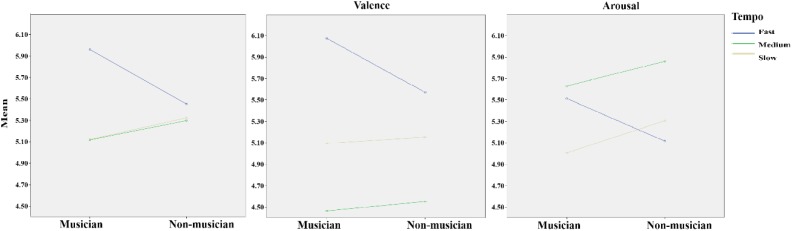
The panel **(Left)** shows the mean scores of musicians’ and non-musicians’ emotional responses to fast, medium, and slow music. The panel **(Middle)** shows the valence scores of musicians’ and non-musicians’ emotional responses to fast, medium, and slow music. The panel **(Right)** shows the arousal scores of musicians’ and non-musicians’ emotional responses to fast, medium, and slow music.

**FIGURE 3:**
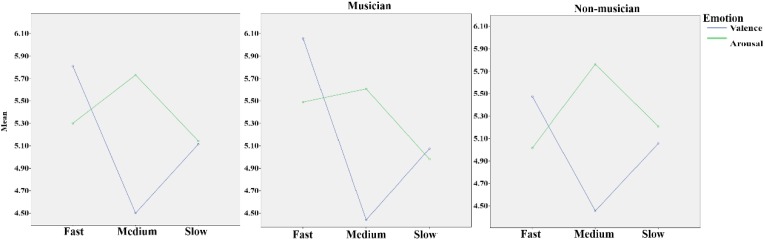
The panel **(Left)** shows the mean scores of all participants’ valence and arousal to fast, medium, and slow music. The panel **(Middle)** shows the mean scores of musicians’ valence and arousal to fast, medium, and slow music. The panel **(Right)** shows the mean scores of non-musicians’ valence and arousal to fast, medium, and slow music.

In the repeated-measure 2 (group: musician and non-musician) × 3 (tempo: fast, medium, and slow) × 2 (emotion dimension: valence and arousal) ANOVA, the main effect of tempo was significant, *F*(2,37) = 5.79, *p* = 0.005, η^2^ = 0.13; the main effect of emotion dimension was significant, *F*(2,37) = 13.57, *p* = 0.001, η^2^ = 0.26. Additionally, the interaction effect of group and tempo approached significance, *F*(2,37) = 2.94, *p* = 0.059, η^2^ = 0.07; the interaction effect of tempo and emotion dimension was significant, *F*(1,37) = 29.38, *p* < 0.001, η^2^ = 0.43. In the one-way ANOVA of group × valence dimension and valence dimension × fast, medium, and slow music, a significant difference was found in the valence of fast music between musicians (6.08 ± 0.84) and non-musicians (5.57 ± 0.72), *F*(1,39) = 4.16, *p* = 0.048, η^2^ = 0.03. Testing the simple effect of rhythm tempo, the valence score of medium-tempo music was significantly lower than those of fast (*p* < 0.001) and slow music (*p* = 0.001); the valence of fast music was significantly higher than that of slow music. The arousal score of medium music was significantly higher than that of fast (*p* = 0.006) and slow music (*p* = 0.003) (Table 1). Overall, musicians perceived more positive and stronger emotions than non-musicians (Figure 2), especially with higher scores of emotional valence in response to fast music. Fast-tempo music had the highest valence and medium-tempo music had lowest valence and highest arousal (Figure 3).

**Table 1:** Average scores of three tempi in two emotional dimensions between musicians and non-musicians.

			Valence			Arousal		
**Tempo**	**Musician**	**Non-musician**	**Musician**	**Non-musician**	**Mean scores of musicians and non-musicians**	**Musician**	**Non-musician**	**Mean scores of musicians and non-musicians**
Fast	5.79 ± 0.56	5.34 ± 0.60	6.08 ± 0.84	5.57 ± 0.72	5.77 ± 0.80	5.51 ± 0.81	5.12 ± 0.76	5.27 ± 0.80
Medium	5.05 ± 0.64	5.21 ± 0.58	4.47 ± 0.79	4.56 ± 0.61	4.52 ± 0.68	5.63 ± 1.05	5.86 ± 0.87	5.77 ± 0.94
Slow	5.05 ± 1.20	5.23 ± 1.13	5.10 ± 1.28	5.16 ± 1.24	5.13 ± 1.24	5.01 ± 1.20	5.31 ± 1.11	5.19 ± 1.14

### fMRI Analyses

#### Group Analysis

**FIGURE 4:**
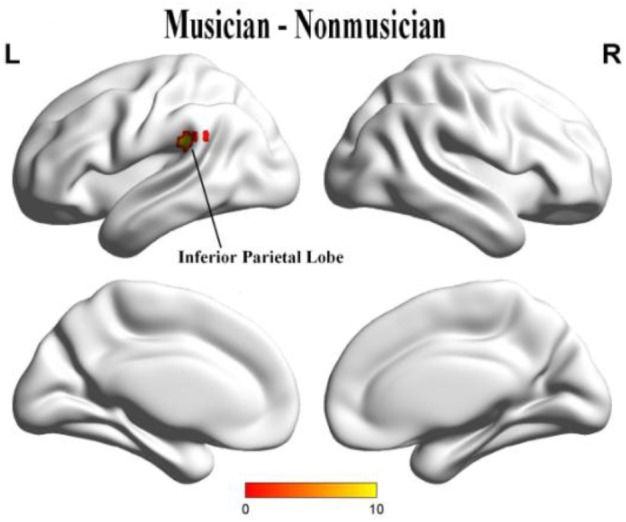
The stronger activation of musicians to non-musicians in left IPL.

When we compared group effects, the musicians demonstrated significantly stronger activation in the left inferior parietal lobe (IPL) than did non-musicians (uncorrected *p* = 0.001, cluster size = 20; Figure 4) (Table 2).

**Table 2:** fMRI analysis of difference between musicians and non-musicians and difference among fast-, medium-, and slow-tempo music.

Condition	Region	Brodmann area	Cluster size	*Z*	*x* (mm)	*y* (mm)	*z* (mm)
Musician – Non-musician							
*P* (uncorrected) < 0.001, cluster size = 20	L_Inferior Parietal Lobe	40	27	3.85	–51	–33	–9
							
Fast–slow							
*P* (FDR correction) < 0.05, cluster size = 20	R_Superior Temporal Gyrus	22	69	4.51	54	–9	3
	L_Superior Temporal Gyrus	22	114	4.08	–57	–18	3
							
Medium–slow							
*P* (FDR correction) < .05, cluster size = 20	R_elsch Gyrus	42	239	4.97	51	–9	6
	R_Precuneus	7	110	4.37	9	–66	33
	R_Middle Temporal Gyrus	21	29	4.27	60	–48	–9
	R_Posterior Cingulate	23	52	4.15	3	–24	30
	R_Inferior Parietal Lobe	40	22	4.14	45	–57	54
	L_Superior Temporal Gyrus	22	152	4.60	–51	–3	0


#### Tempo Analysis

**FIGURE 5:**
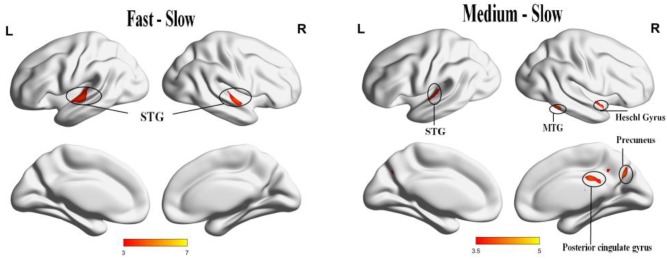
The panel **(Left)** result of comparison shows the positive activation of fast music to slow music in bilateral STG. The panel **(Right)** result of comparison shows the positive activation of medium music to slow music in right HG, right precuneus, right MTG, right PCC, right IPL, left STG, and left culmen.

Significant activation was found when we compared the tempo effects using a *p* < 0.05 cluster-extent FDR correction, cluster size = 20. The activation for fast music was stronger than slow music in the bilateral anterior superior temporal gyrus (STG). The activation for medium music was stronger than slow music in the right Heschl’s gyrus (HG), right precuneus, right middle temporal gyrus (MTG), right posterior cingulate cortex (PCC), right IPL, and left STG (Table 2 and Figure 5).

## Discussion

To our knowledge, this is the first study that provides evidence, from both musical tempo and musical training, to reveal brain activation underlying music-evoked emotion during natural listening conditions. In the current study, the influence of tempo on music-evoked emotion was compared between musicians and non-musicians in their ratings of valence and arousal using music excerpts with fast, medium, and slow tempi. Significant differences were found in emotional valence between musicians and non-musicians when listening to fast music, and in the interaction effect of musical training and tempo. In the functional analysis, musicians showed stronger activation in the left IPL than non-musicians. Both behavioral and neural differences were found among the three tempi, with different activations in the auditory cortex (STG, MTG, and HG), limbic system (cingulate gyrus), and parietal cortex (IPL and precuneus). Especially, when looking at participants’ highest ratings of pleasantness, fast-tempo music showed stronger activation of bilateral STG than did slow music. Medium-tempo music, which participants rated as having the strongest arousal, showed stronger activation in the auditory cortex, cingulate gyrus, and precuneus. Overall, the current results confirmed our hypothesis and provided neural evidence for understanding the effects of musical tempi on musicians and non-musicians’ music experience.

### The Effects of Musical Training

Behaviorally, musicians and non-musicians showed no differences in their integrated emotional ratings of music; however, when the comparison was separated in the valence and arousal dimensions, musicians showed higher emotional valence than non-musicians to fast music. These findings verified our hypothesis that the emotional experience of musicians would be stronger than non-musicians; it also implied that fast-tempo music could effective in differentiating the emotional experience between musicians and non-musicians. The interaction between group and tempo also supported our hypothesis regarding musical training. Participants’ ratings showed a decreasing tendency from musicians to non-musicians in the valence dimension while, in the arousal dimension, there was an increasing tendency from musicians to non-musicians, suggesting that participants’ musical training played a differentiated role in affecting emotional intensity and valence. Further, a significant difference in the neural activation of the IPL was found in the fMRI analysis. A previous study found that only musicians recruited the left IPL for melodic and harmonic processing in a passive fMRI listening paradigm (Schmithorst and Holland, 2003). In their investigation of rhythmic deviation within a musical sequence of musical mismatch negativity, Lappe et al. (2013) also found that bilateral neural activation occurred in the IPL only in musically trained individuals.

The IPL can function in cognitive control with regulating working memory and taking part in observing others’ activities, understanding relevant intentions before beginning subsequent acts with a top-down process (Fogassi et al., 2005). Conversely, we found that the IPL was engaged in responding to emotional information. While viewing emotional images, activation of the IPL has been recognized to arouse the emotions (Ochsner et al., 2012; Ferri et al., 2016). In an emotion-detecting experiment, Engelen et al. (2015) presented neutral and emotional body conditions of equaled motion; they found stronger activation of the IPL in the emotional condition in contrast to a neutral body gesture. The left IPL plays a pivotal role in many cognitive functions (Bzdok et al., 2016), such as second language learning (Sliwinska, 2015; Barbeau et al., 2017), semantic processing (Patel, 2003; Donnay et al., 2014), auditory working memory (Alain et al., 2008), and implicit processing of brief musical emotions (Bogert et al., 2016). In the magnetoencephalography (MEG) recording of cortical entrainment to music and its modulation by expertise, left lateralization was confirmed in a topographical analysis of the difference between musicians and non-musicians in the beta range (Doelling and Poeppel, 2015). Combined with the higher valence of musicians to non-musicians, the stronger activation of the IPL could be important evidence of superior processing for musicians’ emotional experience.

Some studies have reported than the emotional response to music was stable between musicians and non-musicians and was weakly influenced by musical expertise (Bigand et al., 2005; Bramley et al., 2016). However, in their experiments, musicians and non-musicians were both asked to perform other tasks, such as to identify excerpts that induced similar emotional experiences and to group these excerpts or involve participants in a virtual roulette paradigm. These scenarios are different from the current study’s design of no-task music listening for emotional experience, which is close to the music listening situation in people’s daily life. As a natural musical listening study of music emotion evoking, the present findings highlight the differences between musicians and non-musicians, and provided a further understanding of humans’ music experience.

### The Effect of Rhythm Tempo

#### Fast Music vs. Slow Music

Consistent with our hypothesis, fast-tempo music evoked the most pleasant emotion, and slow-tempo music received the lowest score in the arousal dimension among the three kinds of music. These findings are consistent with existing research about music tempo and its influence on subjective emotional valence and arousal (Droitvolet et al., 2013; Trochidis and Bigand, 2013). Happy music produced significant and large activation in the auditory cortices (Brattico et al., 2011; Koelsch et al., 2013; Park et al., 2014; Bogert et al., 2016). The STG is the core region of the auditory area that responds to auditory stimuli (Humphries et al., 2010; Angulo-Perkins et al., 2014; Thomas et al., 2015) and has also been characterized for happy compared with sad music conditions (Pehrs et al., 2013; Bogert et al., 2016). The current fMRI results, that bilateral STG was stronger for fast- than slow-tempo music were consistent with previous findings, which implied that a faster tempo could arouse more positive emotion with stronger activation of emotional experience in the temporal cortex.

However, this finding was different from a previous study, in which no changes in musical tempo were found in humans’ autonomic nervous system (ANS) when participants listened to fast and slow music (Krabs et al., 2015). Although music-evoked emotion had been reported to contribute to music-evoked ANS effects (Juslin, 2013) and it was tightly connected with music tempo. In the study of Krabs et al. (2015), the tempo was extracted after the musical emotion was identified as pleasant or unpleasant and ANS effects did not differ between fast music and slow music, which implied that the neural effects of musical tempo could not be equaled to musical emotion. In our study, we chose the tempo to be the core variable to compare its emotional effects and emotional valence was the second variable, which depended on the chosen tempo. Compared with the above study, the different STG activation of tempo in our research may be more objective to explain that tempo was an acoustical feature that affected listeners’ emotional experience and blood oxygenation level dependent (BOLD) response, but not a transitional emotion label in music; alternatively, it can be explained that fMRI activities were more sensitive than ANS response affected by tempo. Conclusively, conjunct findings of higher emotional valence and bilateral STG that were stronger in fast- than slow-tempo music were both produced in the current study, which provided a powerful explanation of the effects of musical tempo on humans’ emotional experience.

#### Medium Music vs. Slow Music

Although it was beyond our expectation that participants rated medium-tempo music with the strongest arousal and the lowest valence in this study, a large range of neural activation in the auditory cortex, PCC, precuneus, and IPL could provide a scientific explanation for this understanding. The cingulate gyrus is an important structure in the core music-evoked emotion network (Koelsch, 2014) and was also found to be positively connected with humans’ ability to regulate emotions after long-time music exposure (Bringas et al., 2015). The PCC is believed to link emotion and memory processes (Maratos et al., 2001; Maddock et al., 2003) and has been implicated in autobiographical emotional recall (Fink et al., 1996). The precuneus is another important area for feeling the emotional content of music (Blood and Zatorre, 2001; Tabei, 2015), and is a brain region associated with assessing emotional responses evoked in the listener (Cavanna and Trimble, 2006). Through large functional analysis of postmortem assessments of cingulotomy lesions, vegetative state cases, and metabolic studies, the PCC and precuneus have been proven to be pivotal for conscious information and connected with arousal state (Vogt and Laureys, 2005). Regarding medium-tempo music demonstrating the strongest arousal in this study, it could be concluded that these high levels of emotional arousal may be tightly connected with listeners’ PCC and precuneus. Participants rated medium music as having the lowest emotional valence, which means they felt the least pleasure or weakest hedonic component (Feldman, 1995). The right hemisphere has been shown to be more connected with negative emotion (Sato and Aoki, 2006). When medium music was contrasted with slow music, the temporal cortex, cingulate gyrus, and precuneus were both activated in the right hemisphere, which was connected to the weak pleasure recorded for medium music.

In addition to the limbic system (cingulate gyrus) and parietal cortex (IPL and precuneus), significant neural difference was also found in the auditory cortex (HG, MTG, and STG) when medium music was contrasted with slow music. HG is an area of the primary auditory cortex buried within the lateral sulcus of the human brain (Dierks et al., 1999). It plays an important role in pitch perception and initial detection of acoustic changes (Krumbholz et al., 2003; Schneider et al., 2005). Activation of the right MTG has been found to be connected to the processing of semantic aspects of language (Hickok and Poeppel, 2007), and music-evoked emotion by expressed affect (Steinbeis and Koelsch, 2008). Even in a distorted tune test, the right MTG was found to be stronger in musicians with 19 years of training than in individuals without musical training (Seung et al., 2005). Medium music aroused a similar BOLD signal of MTG in listeners with music training, which provided a possible explanation that medium music would be more readily available for the listeners’ acoustic perception. We found that medium-tempo music only elicited stronger STG activation in the left hemisphere. The auditory cortices in the two hemispheres are relatively specialized. Specifically, temporal resolution is greater in the left auditory cortical areas and spatial resolution is greater in the right auditory cortical areas (Zatorre et al., 2002; Liu et al., 2017). Studies have found that trained music listeners showed stronger activation in the left STG during passive music listening (Ohnishi et al., 2001; Bangert et al., 2006). Finding this activity in the left STG inspired us to theorize that medium music may promote individuals’ music processing with the left hemisphere’s advantage in temporal resolution. The current results demonstrated that music with a medium tempo could promote listeners’ emotional arousal through a large range of neural activities. Whether it depends on the similarity of medium-tempo music to human physiological rhythms (i.e., the rhythm of the heart), or other internal neural mechanisms, deserves future investigation combined with more biological traits and technology.

## Limitation and Future Studies

Our study addressed the effects of musical tempo on music-evoked emotion and how it is influenced by humans’ musical training. Although no motor areas were found in the current study, the experiment described new neural characteristics of the mechanism of tempo on music-evoked emotion. There are two limitations in the current study. No significant relationships were found between the behavioral scores and the BOLD signals. In the current study, the emotional ratings of music were collected after scanning to ensure an unpolluted and natural music listening situation. In future study, the data collection of emotional dimensions and BOLD signals may be improved with an effective method of integration. Besides, only three kinds of music tempo were presented to compare their effects on music-evoked emotion. If stimuli can be used in a continuous fashion, more relationships between these variables may be found in future researches.

## Summary

Tempo is highly connected with music-evoked emotion and it interacts with musical training during behavioral tasks. This study discussed the emotional experience with valence and arousal ratings and explained the neural findings of fast-, medium-, and slow-tempo music. The stronger activation of the IPL in musicians than non-musicians provides supplementary evidence for the controversial conclusion of whether or not musical training promotes listeners’ neural activation to emotional music. We synthesized key findings about the auditory area, parietal cortex, and cingulate gyrus to explain the highest valence to fast music and the strongest arousal to medium music. Although no significant interactions between musical training and tempo were found in the fMRI analysis, the behavior interactions provided a worthy inspiration for future investigation. Hence, the effects of tempo on music-evoked emotion could be a valuable investigation for revealing the emotional mechanism in listening to music.

## Author Contributions

GL was the leader of this study and instructed the study theme. YL was responsible for the experimental design and manuscript writing. DW participated in experimental design. QL, GY, and SW were responsible for the data collection. GW and XZ were responsible for the recording the stimuli and selecting the music stimuli.

## Conflict of Interest Statement

The authors declare that the research was conducted in the absence of any commercial or financial relationships that could be construed as a potential conflict of interest. The reviewer NG and handling Editor declared their shared affiliation at time of review.

## Footnotes

1 https://www.fil.ion.ucl.ac.uk/spm/software/spm8/
