# Supplementary material for: Effects of Musical Tempo on Musicians’ and Non-musicians’ Emotional Experience When Listening to Music
Source: Front Psychol. 2018 Nov 13;9:2118. doi: 10.3389/fpsyg.2018.02118 (PMC6243583; doi:10.3389/fpsyg.2018.02118)
Supplement: Supplementary file 1 [file Table_1.PDF]

Table 1S. List of experimental music materials

		Music	Version	Tempo
Fast	1	The first movement of Yellow River Piano Concerto	Chengzong Yin	Allegro
	2	Chapter IV of Zigeunerweisen op.20	Jascha Heifetz	Allegro
	3	Chopin: Grande Valse Brillante in E-Flat Major, Op. 18, No. 1	Langlang	Allegro
	4	The Flight of the Bumble-Bee	Maksim Mrvica	Presto
Medium	1	Fishermen's Song at Eventide	China National Symphony Orchestra	Moderato
	2	M á Vlast (My Country) - II. Vltava (The Moldau)	Israel Philharmonic Orchestra	Andante
	3	Morgenstemning	Edvard Grieg	Moderato
	4	Butterfly Lovers' Violin Concerto	Wei Xue	Andante
Slow	1	The Palm-leaf Beated By Raindrop	China Broadcasting Chinese Orchestra	Larghetto
	2	Moonlight sonata	Richard Clayderman	Adagio
	3	Traumerei	Robert Schumann	Larghetto
	4	Frontlan Ling Wang into Song	Television Music of The Empress of China	Larghetto
